# Two new species of the *Aenictus wroughtonii* species group (Hymenoptera, Formicidae, Dorylinae) from Thailand

**DOI:** 10.3897/zookeys.775.26893

**Published:** 2018-07-19

**Authors:** Weeyawat Jaitrong, Jiraporn Ruangsittichai

**Affiliations:** 1 Natural History Museum, National Science Museum, Technopolis, Khlong 5, Khlong Luang, Pathum Thani, 12120 Thailand; 2 Department of Medical Entomology, Faculty of Tropical Medicine, Mahidol University, 420/6 Ratchawithi Road, Ratchathewi, Bangkok, 10400 Thailand

**Keywords:** *Aenictus wroughtonii* species group, *Aenictus minutulus* species group, army ant, distribution, new species, taxonomy, Thailand

## Abstract

The *Aenictus wroughtonii* species group is widely distributed in Asia. The members of this group are characterised by a slender body, long legs, anterior clypeal margin with 5–10 denticles and a weakly-developed subpetiolar process. Twelve worker-based species of this group have been recorded from Asia. Herein, two new species from Thailand (*Aenictus nuchiti* **sp. n.** and *Aenictus samungi* **sp. n.**) are added to this group. A key to the Asian species of this group is provided.

## Introduction

The *Aenictus wroughtonii* species group was established by Wilson (1964) based on the external morphology of the worker caste. Subsequently, Jaitrong et al. (2010) and Jaitrong and Yamane (2011) redefined the species group and listed seven worker-based species from the Oriental region. Recently, Sharaf et al. (2012) and Staab (2014) described new species of the group from Saudi Arabia and Southeast China, respectively. To date, 10 species have been recorded in the *A. wroughtonii* group from Greece, Turkey, Iran, Israel, India, Sri Lanka, Southeast China and Taiwan to Southeast Asia (Aktaç et al. 2004, Jaitrong et al. 2010, Jaitrong and Yamane 2011, Sharaf et al. 2012, Staab 2014).

During our survey of the Asian *Aenictus*, two unidentified species belonging to the *A. wroughtonii* group were found from Thailand. After carefully examining specimens of these two species under a stereomicroscope and comparing them with the type material of closely related species, it was concluded that both species are new to science. In the present study, the two new species are described and a key for the Asian species based on the worker caste is provided.

## Materials and methods

The holotypes and paratypes of *Aenictus nuchiti* sp. n. and *Aenictus samungi* sp. n. were pin-mounted dry specimens. The holotypes, paratypes and syntypes of seven species (*A. artipus* Wilson, 1964; *A. biroi* Forel, 1907; *A. camposi* Wheeler WM & Chapman, 1925; *A. sagei* Forel, 1901; *A. stenocephalus* Jaitrong & Yamane, 2010; *A. vieti* Jaitrong & Yamane, 2010; *A. wroughtonii* Forel, 1890) in the *A. wroughtonii* species group were examined. High-resolution images of the holotype of *A. gutianshanensis* Staab, 2014 available in Antweb (2018) were also examined. Most morphological observations were made with a ZEISS Discovery V12 stereoscope.

Multi-focused montage images were produced using NIS-Elements-D-[Sequence6*-Focused] from a series of source images taken by a Nikon Digital Sight-Ri1 camera attached to a Nikon AZ100M stereoscope. Type specimens of each species were measured for the following parts using a micrometer (accurate to 0.01 mm).

The abbreviations used for the measurements and indices are as follows:

**HL** Maximum head length in full-face view, measured from the anterior clypeal margin to the midpoint of a line drawn across the posterior margin of the head.

**HW** Maximum head width in full-face view.

**ML** Mesosomal length measured from the point at which the pronotum meets the cervical shield to the posterior margin of the metapleuron in profile.

**PH** Petiolar node height, measured in profile, the maximum vertical height of the petiole from summit to lower most part of subpetiolar process.

**PL** Petiole length measured from the anterior margin of the peduncle to the posteriormost point of the tergite in profile.

**SL** Scape length excluding the basal constriction and condylar bulb.

**TL** Total length, axial length of body, summed HL (including mandibles) + ML + PL + postpetiole length + gaster length.

**CI** Cephalic index, HW/HL × 100.

**PI** Petiolar index, PH/PL × 100.

**SI** Scape index, SL/HW × 100.

Abbreviations of the type depositories are as follows:

**MHNG** Muséum d’histoire naturelle, Geneva, Switzerland

**SKYC** Seiki Yamane Collection, Japan

**THNHM** Natural History Museum of the National Science Museum, Thailand

**USNM** National Museum of Natural History, Smithsonian Institution, Washington DC, USA

The general terminology for the worker caste of the ants follows Hölldobler and Wilson (1990) and Bolton (1994). The studies of Jaitrong et al. (2010) and Jaitrong and Yamane (2011) have been referred to for the important characteristics of the genus *Aenictus*.

## Results

### Taxonomy

#### Aenictus wroughtonii

Taxon classificationAnimaliaHymenopteraFormicidae

species group

Figs 1A , 3A

##### Diagnosis.

**Figure 1.**
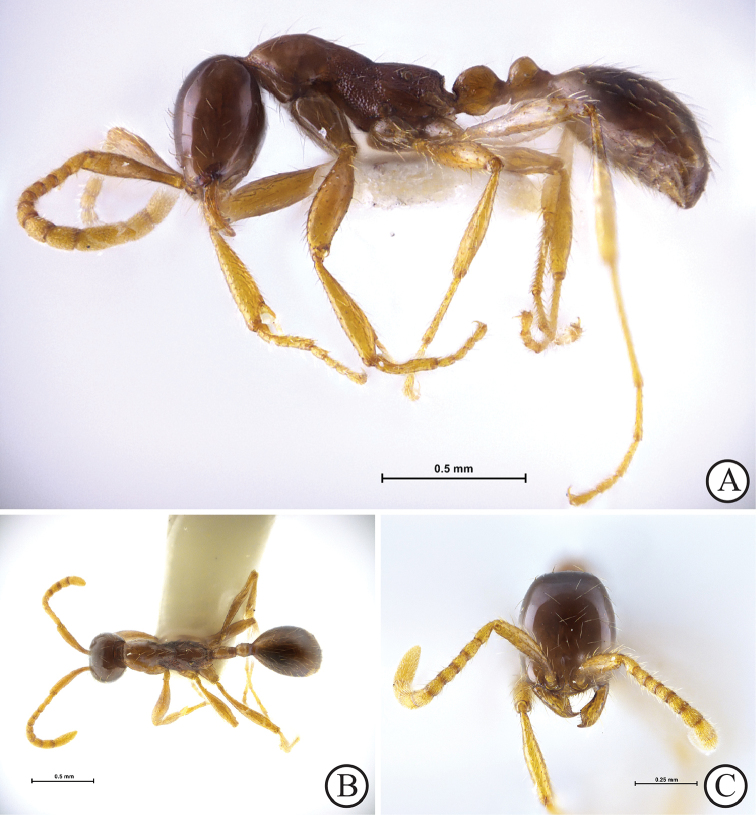
*Aenictus nuchiti* sp. n. (holotype, worker, THNHM-I-02612). **A** Body in profile **B** Body in dorsal view **C** Head in full-face view.

(modified from Jaitrong and Yamane 2011). Head narrow, oval or elliptical; occipital margin lacking collar (distinct carina). Antenna short or long, comprising 10 segments; scape short, attaining mid-length of head or longer attaining or extending beyond the posterolateral corner of head. Anterior clypeal margin roundly convex with 5–10 denticles. Mandible triangular, with masticatory margin bearing 8–12 minute inconspicuous denticles in addition to a large apical tooth with a sharp apex; basal margin of mandible lacking denticles. Frontal carina short; parafrontal ridge feeble and incomplete. Mesosoma narrow and elongate. Legs very slender. Propodeal junction in profile angulate (Fig. 1A) or rounded (Fig. 3A). Subpetiolar process weakly developed or almost absent. Head and gaster entirely smooth and shiny. Body yellow, yellowish brown to dark brown; typhlatta spot absent.

##### Distribution.

Greece, Iran, Israel, Turkey, Saudi Arabia, India, Sri Lanka, Southeast China, Taiwan, Vietnam, Thailand, Malay Peninsula (West Malaysia), Sumatra, Borneo (Sabah, Sarawak, and Brunei) and Philippines (Negros and Luzon) (Aktaç et al. 2004, Jaitrong et al. 2010, Jaitrong and Yamane 2011, Sharaf et al. 2012, Staab 2014).

##### Currently valid names.

*Aenictus arabicus* Sharaf & Aldawood, 2012, *A. artipus* Wilson, 1964; *A. biroi* Forel, 1907; *A. camposi* Wheeler WM & Chapman, 1925; *A. gutianshanensis* Staab, 2014, *A. nuchiti* sp. n., *A. rhodiensis* Menozzi, 1936, *A. sagei* Forel, 1901; *A. samungi* sp. n., *A. stenocephalus* Jaitrong & Yamane, 2010; *A. vieti* Jaitrong & Yamane, 2010; *A. wroughtonii* Forel, 1890.

#### Descriptions of new species

##### Aenictus nuchiti sp. n.

Taxon classificationAnimaliaHymenopteraFormicidae

http://zoobank.org/C0440537-2B93-4EA6-8A29-0D7CACEB3ABB

Figs 1 , 2 , 4

###### Types.

Holotype (THNHM-I-02612, THNHM), 55 paratype workers (THNHM-I-02614, MHNG, SKYC, THNHM, USNM) and queen (THNHM-I-02613, THNHM), N Thailand, Chiang Mai Province, Omkoi District, Omkoi Forest, DDF (dry dipterocarp forest), 17.89583333°'N, 98.40750000°E, ca 1000 m a.s.l., 16.VII.2016, W. Jaitrong leg., Colony no. TH16-WJT-859.

###### Non-type material examined.

28 workers, Thailand, Chiang Mai Province, San Sai District, San Sai Luang Sub-district, near Maejo University Campus, mixed deciduous forest, collected from leaf litter, 18.92777778°N, 99.05083333°E, ca 350 m a.s.l., 15.X.2015, N. Likhitrakarn leg., Colony no. NL151015-1 (THNHM).

###### Worker measurements.

Holotype: HL 0.53; HW 0.40; ML 0.69; PH 0.17; PL 0.18; SL 0.41; TL 2.28; CI 75; PI 91; SI 104. Paratype workers (n = 10): HL 0.50–0.53; HW 0.38–0.43; ML 0.66–0.73; PH 0.13-0.17; PL 0.17–0.18; SL 0.40–0.43; TL 2.24–2.41; CI 77–83; PI 90-91; SI 94–104.

###### Queen measurements.

(paratype). HL 0.92; HW 01.06; ML 1.55; PH 0.53; PL 0.53; SL 0.64; TL 5.31; CI 114; PI 100; SI 61.

###### Description of Worker.

(Holotype and paratypes; Fig. 1). Head in full-face view elliptical, clearly longer than broad with slightly convex sides and almost straight posterior margin. Antennal scape short, extending beyond the mid-length of the head but not reaching the posterolateral corner of the head; antennal segment II slightly longer than III–VI; the last (X) almost as long as VIII and IX combined and as long as II and III combined. Frontal carina thin and short, not extending beyond level of posterior margin of torulus. Clypeus short, with its anterior margin roundly convex, bearing 7 denticles. Mandible with an apical tooth large and curved, followed by a medium-sized subapical tooth and a series of 10–12 minute teeth on masticatory margin. Mesosoma in profile with pronotum strongly convex dorsally, demarcated from mesonotum by a shallow transverse groove; mesonotum convex, sloping gradually to metanotal groove; mesopleuron demarcated from metapleuron by a shallow groove. Propodeum in profile lower than promesonotum, with a weakly convex dorsal outline; propodeal junction angulate; declivity of propodeum widely and shallowly concave, encircled by a thin rim. Petiole in profile slightly longer than high, with a dorsal outline convex; seen from above relatively narrow with sides almost parallel; subpetiolar process present, its ventral outline convex, without angle or tooth; postpetiole slightly shorter than petiole but seen from above slightly broader than petiole; its node short, clearly shorter than high.

Head, antennal scapes, pronotum, petiole, postpetiole, gaster, femora and tibiae of legs entirely or extensively smooth and shiny. Antennal flagellum densely punctate; mesothorax and propodeum with dense punctures; metapleuron partly or extensively smooth.

Body with relatively sparse standing hairs mixed with sparse short hairs over surface; longest pronotal hair 0.10–0.13 mm long. Head, mesonotum, propodeum and gaster dark brown; pronotum, waist, antennae and legs reddish brown.

###### Description of Queen.

**Figure 2.**
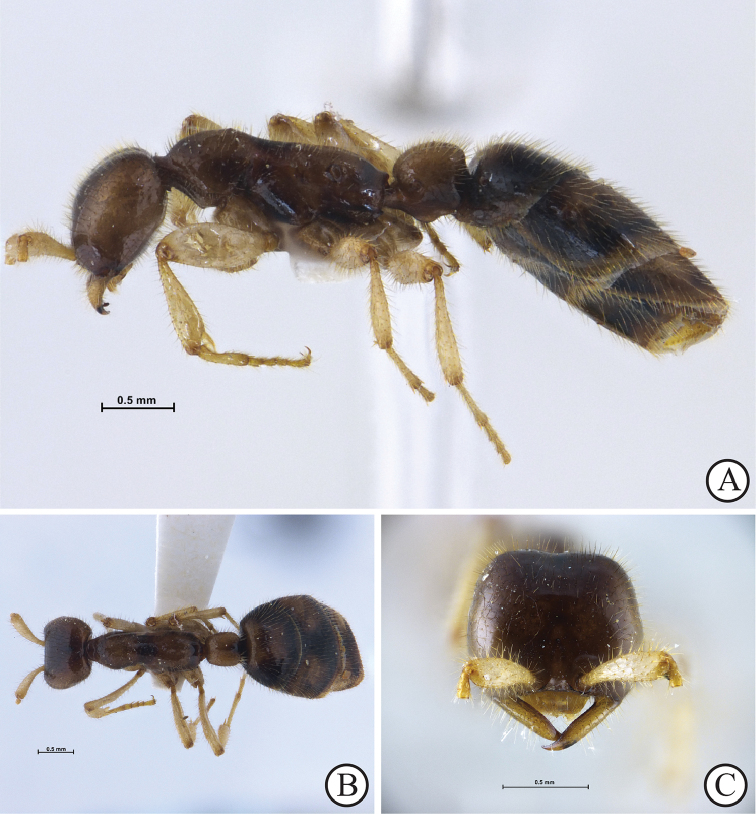
*Aenictus nuchiti* sp. n. (paratype, queen, THNHM-I-02613). **A** Body in profile **B** Body in dorsal view **C** Head in full-face view.

(Paratype, Fig. 2). Head in full-face view subrectangular, posteriorly narrow and gradually widening anteriorly, slightly shorter than broad, with sides weakly convex and posterior margin concave; upper frons weakly concave. Antennal scape flat, relatively short, about half as long as head, basally narrow, widening considerably apicad; flagellum of antenna missing (for this specimen). Frontal carina indistinct. Parafrontal ridge absent. Anterior clypeal margin concave, without denticles. Mandible half as long as head length, with a slender, inner margin that is convex while lateral margin weakly concave; masticatory margin without denticles. Mesosoma elongate; in profile, pronotum convex dorsally; mesonotum weakly concave; propodeal dorsum almost straight; seen from above pronotum and propodeum broader than mesonotum; propodeal junction low, roundly convex; propodeal declivity weakly convex, not encircled by a rim. Petiole longer than high, with its dorsal outline slightly elevated posteriorly, with petiole in profile posterodorsal corner bluntly angulate; seen from above petiole with a distinct longitudinal furrow running from anterior face to posterior face; subpetiolar process large, subtriangular, with its apex pointed downwards. Gaster large and elongate; first tergite narrower and shorter than second, its anterior slope weakly concave; second tergite largest; third as long as first; tip of gaster missing in this specimen. Legs relatively long and slender; femora and tibiae clavate.

Entire body smooth and shiny, with relatively dense standing hairs; hairs slightly shorter on pronotum than on head, mandible and antennal scape; longest pronotal hair 0.08 mm long. Head dark brown; lateral and ventral faces of head and mandible reddish brown; scapes and legs yellowish brown. Mesosoma with ground colour reddish brown; lateral faces of pronotum and mesonotum, entire mesopleura and propodeal declivity dark brown. Petiole with ground colour reddish brown; lower portion of petiole, posterior slope of petiole and subpetiolar process dark brown; gaster with ground colour dark brown; lateral faces of second tergite reddish brown.

###### Etymology.

The species is named after Mr Supachai Nuchit (Royal Forest Department, Thailand) who kindly helped us with ant collecting at Pa Omkoi National Forest, Chiang Mai Province.

###### Distribution.

Northern Thailand (Chiang Mai Province).

###### Comparative diagnosis.

*Aenictus nuchiti* sp. n. is most similar to *A. biroi*, *A. camposi*, *A. gutianshanensis* and *A. vieti* in having dense punctures on the mesosoma and an angulate propodeal junction. However, *A. nuchiti* is much smaller than the latter four (TL 2.24–2.41 mm, HW 0.38–0.43 mm in *A. nuchiti*; TL > 2.6 mm, HW > 0.43 mm in the latter four). It has a short antennal scape that reaches only two-thirds the head length (in contrast, reaching or extending beyond the posterolateral corners of the head in the latter four). This species can be distinguished from *A. gutianshanensis* and *A. vieti* by the configuration of the subpetiolar process (ventral outline roundly convex and without anterior angle in *A. nuchiti*; ventral outline with anterior angle in *A. biroi*, *A. gutianshanensis* and *A. vieti*). *Aenictus nuchiti* is similar to *A. biroi* and *A. camposi* in the unarmed subpetiolar process. In *A. nuchiti*, however, the body size is much smaller than that of *A. biroi* and the head is clearly longer than broad in *A. nuchiti* (almost as long as broad in *A. biroi*). The body colour is dark brown in *A. nuchiti*, whereas it is entirely yellow in *A. camposi*. The propodeal declivity is broader and widely rounded above in *A. nuchiti* but is narrow and tapers distinctly above in *A. camposi*.

##### Aenictus samungi sp. n.

Taxon classificationAnimaliaHymenopteraFormicidae

http://zoobank.org/758F945A-D992-438B-ACFC-BD8E55283AB6

Figs 3 , 5

###### Types.

Holotype (THNHM-I-02615, THNHM) and 15 paratype workers (THNHM-I-02616, MHNG, SKYC, THNHM, USNM), Thailand, Tak Province, Um Phang District, Thung Yai Wildlife Sanctuary, Yuyi Junction, DEF (dry evergreen forest), 15.44861111°N, 99.04694444°E, ca 1100 m a.s.l., 25.IX.2016, W. Jaitrong leg., TH16-WJT-1069.

###### Non-type material examined.

One worker, Thailand, Tak Province, Near Myanmar border, Tung Yai [Thung Yai] W.S., 23.V.1999, W. Jaitrong leg. (THNHM).

###### Measurements.

Holotype: HL 0.41; HW 0.31; ML 0.53; PH 0.17; PL 0.12; SL 0.20; TL 1.75; CI 76; PI 143; SI 63. Paratypes (n = 11): HL 0.40–0.43; HW 0.31–0.33; ML 0.51–0.53; PH 0.17–0.18; PL 0.12–0.13; SL 0.20–0.23; TL1.72–1.78; CI 76–80; PI 138–143; SI 63–70.

###### Description of Worker.

**Figure 3.**
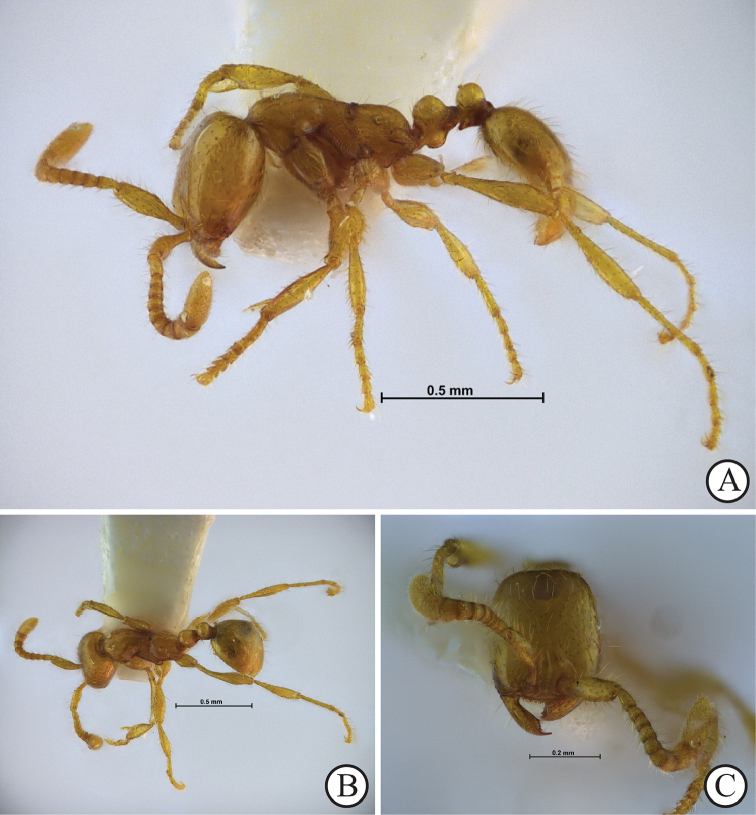
*Aenictus samungi* sp. n. (holotype, worker, THNHM-I-02615). **A** Body in profile **B** Body in dorsal view **C** Head in full-face view.

(holotype and paratypes; Fig. 3). Head in full-face view clearly longer than broad, with its sides weakly convex and posterior margin almost straight or feebly concave. Antennal scape short, only slightly extending mid-length of head; antennal segment II (pedicel) clearly longer than each of III–VI; X longer than VII, VIII and IX combined. Frontal carinae fused at level of anterior margin of torulus, extending beyond level of posterior margin of torulus. Clypeus short, with its anterior margin bearing 7 denticles. Mandible subtriangular, with apical tooth large and curved, followed by a medium-sized subapical tooth, 4–5 minute teeth and a medium-sized basal tooth on masticatory margin. Mesosoma in profile almost flat dorsally; metanotal groove distinct. Propodeum in profile with a nearly straight dorsal outline; propodeal junction rounded; propodeal declivity weakly convex, not encircled by a rim. Petiole including subpetiolar process shorter than high, with its dorsal outline convex; subpetiolar process rather developed, with its ventral outline roundly convex, without angle or tooth; postpetiole shorter than petiole and shorter than high, in profile slightly elevated posteriorly.

Head, antennal scape, promesonotum, propodeal dorsum, petiole, postpetiole, gaster, femora and tibiae of legs entirely or extensively smooth and shiny; mesopleuron, metapleuron and lateral face of propodeum superficially reticulate. Antennal flagellum densely finely punctate.

Body with relatively sparse standing hairs mixed with sparse short hairs over surface; longest pronotal hair 0.07–0.08 mm long. Head, mesosoma, waist, gaster, antennae and legs yellowish brown; mandible dark brown.

###### Etymology.

The species is named after Mr Yuthana Samung (Faculty of Tropical Medicine, Mahidol University, Thailand) who kindly helped us in taking pictures of Thai ants, including the two new species discovered in the present study.

###### Distribution.

Western Thailand (Tak Province).

###### Comparative diagnosis.

*Aenictus samungi* sp. n. can be easily distinguished from the other members of the *A. wroughtonii* species group by the following characteristics: smallest species of the group (HW 0.31–0.33 mm in *A. samungi*; HW > 3.7 mm in other members); petiole shorter than high (as long as or longer than high in other members); promesonotum with almost flat or straight dorsal outline (convex and sloping gradually to metanotal groove in other members); antennal scape short only just reaching mid-length of the head (at least two-thirds of the head length or beyond the posterolateral corner of the head in other members). Both *Aenictus nuchiti* sp. n. and *A. samungi* sp. n. have small bodies and short antennae, but can be easily separated from each other by the different conditions of the propodeum (Figs 1A vs. 3A).

#### Key to Asian species of the *Aenictus wroughtonii* species group based on worker caste

**Table:** 

1	Antennal scapes short, when laid back only attaining mid-length or two-thirds of the head length	**2**
–	Antennal scape long, when laid back attaining or extending beyond the posterolateral corner of the head	**4**
2	HW 0.31–0.33 mm; antennal scape attaining only mid-length of the head; petiole excluding subpetiolar process shorter than high (Thailand)	***A. samungi* sp. n.**
–	HW > 0.37 mm; antennal scape attaining two-thirds of the head length; petiole excluding subpetiolar process longer than high	**3**
3	Propodeal junction rounded; lateral faces of the petiole and postpetiole superficially reticulate; subpetiolar process present, its anteroventral corner angulate; postpetiole slightly longer than the petiole (Saudi Arabia)	***A. arabicus***
–	Propodeal junction angulate; lateral faces of the petiole and postpetiole entirely smooth and shiny; subpetiolar process convex dorsally, anteroventrally not angulate; postpetiole slightly shorter than the petiole (Thailand)	***A. nuchiti* sp. n.**
4	Propodeal junction rounded	**5**
–	Propodeal junction angulate	**9**
5	Subpetiolar process almost absent, anteroventrally not angulate (India)	***A. wroughtonii***
–	Subpetiolar process weakly developed; its anteroventral corner angulate	**6**
6	Entire pronotum and petiole sculptured (punctate or reticulate) (China)	***A. gutianshanensis***
–	Pronotum and petiole largely smooth and shiny	**7**
7	HW 1.00–1.04 mm (Aktaç et al. 2004, measurements of syntypes); posterior margin of head concave (Greece, Iran, Israel, Turkey)	***A. rhodiensis***
–	HW < 0.60 mm; posterior margin of head almost straight or convex	**8**
8	Scape short; SI 100 or less; body hairy; the longest pronotal hair 0.23–0.25 mm; subpetiolar process very low, with ventral outline almost straight (India, Nepal, Afghanistan)	***A. sagei***
–	Scape long; SI 130–140; body with sparse hairs; the longest pronotal hair approximately 0.15–0.18 mm; subpetiolar process with ventral outline slightly convex (China, Vietnam, Thailand)	***A. artipus***
9	Ventral outline of subpetiolar process convex, anteroventrally not angulate	**10**
–	Ventral outline of subpetiolar process convex or almost straight; its anteroventral corner angulate	**11**
10	Declivity of propodeum narrower, seen from back strongly tapering above; petiole longer than high (PI 84–86); body smaller with TL 2.6–2.7 mm; antenna longer with SI 122–135 (Thailand, Indonesia, Malaysia, Philippines)	***A. camposi***
–	Declivity of propodeum broader and more rounded above; petiole almost as long as high (PI 95–100); body larger with TL 3.1–3.2 mm; antenna shorter with SI 114–118 (Sri Lanka)	***A. biroi***
11	Ventral outline of subpetiolar process strongly convex in anterior half; mesonotum partly and propodeum almost entirely densely sculptured; pronotum clearly demarcated from mesonotum by a shallow transverse groove (Taiwan, Vietnam and Thailand)	***A. vieti***
–	Ventral outline of subpetiolar process almost straight; entire mesonotum and propodeum smooth and shiny, with at most superficial sculpture; pronotum only weakly demarcated from mesonotum (Thailand)	***A. stenocephalus***

## Discussion

These two new species are also similar to members of the *Aenictus minutulus* species group (*A. changmaianus* Terayama & Kubota, 1993, *A. minutulus* Terayama & Yamane, 1989, *A. minimus* Jaitrong & Hashimoto, 2012) in general appearance and by having a short petiole, short antennal scapes (reaching only to mid-length of the head) and subtriangular mandibles (masticatory margin with a large apical tooth, medium-sized subapical and basal teeth and 2–6 denticles between them) (Jaitrong and Hashimoto 2012). However, herein, both species were treated as members of the *A. wroughtonii* group because they have a serrate anterior clypeal margin, the most important characteristic that separates the *A. wroughtonii* group from the *A. minutulus* group (anterior clypeal margin without denticles in the *A. minutulus* group) (see the key for the species groups of *Aenictus* in Jaitrong and Yamane (2011)).

**Figure 4.**
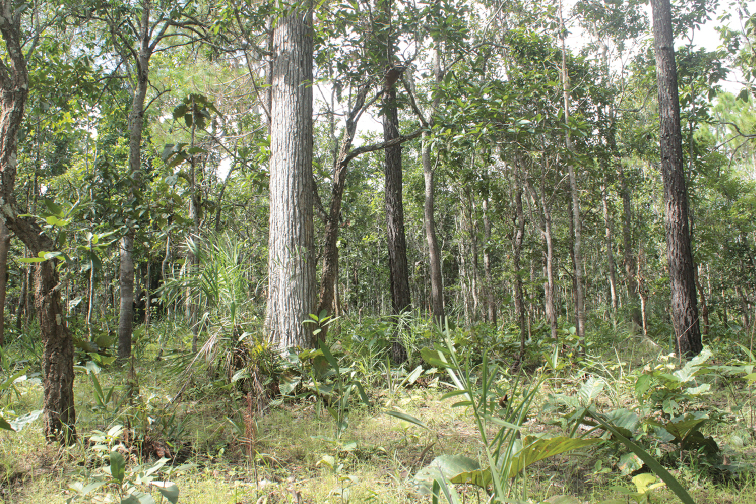
Type locality of *Aenictus nuchiti* sp. n., dry dipterocarp forest in Chiang Mai Province, northern Thailand.

*Aenictus nuchiti* sp. n. is a rare species. The type series was collected from a bivouac under a large rotting log in a dry dipterocarp forest (Fig. 4), ca 1000 m a.s.l., during the wet season. Numerous immature specimens (all were pupae) were found in the bivouac, and no worker activity was seen around the log. Another colony (NL151015-1) was collected from a leaf litter in a dry dipterocarp forest, ca 350 m a.s.l. This new species is sympatric with *A. artipus* belonging to the same species group in at least the Chiang Mai Province (Wilson 1964; Jaitrong et al. 2010; Jaitrong and Yamane 2011).

**Figure 5.**
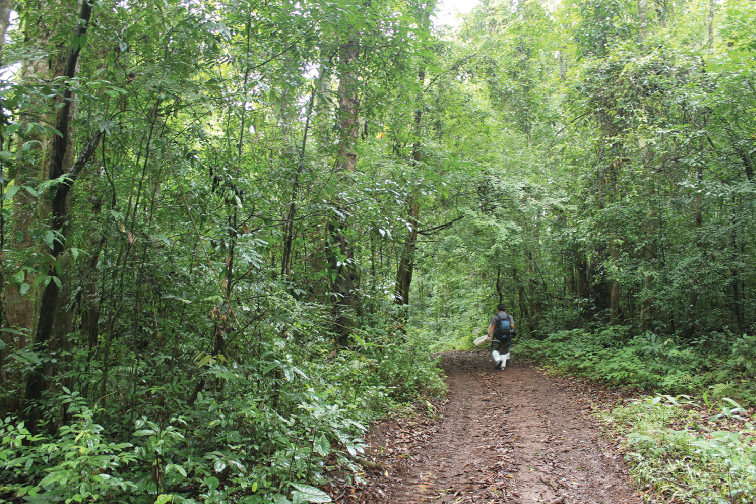
Type locality of *A. samungi* sp. n., dry evergreen forest in Tak Province, western Thailand.

*Aenictus samungi* sp. n. is also a rare species and is known only from the type locality (ca 1100 m a.s.l.). The type series was collected from a foraging column on a forest path in a dry evergreen forest (Fig. 5), western Thailand near the Myanmar border; no immature and prey were seen along the column. Workers were fast-running.

## Supplementary Material

XML Treatment for Aenictus wroughtonii

XML Treatment for Aenictus nuchiti

XML Treatment for Aenictus samungi
